# Sublethal effects of a commercial Bt product and Bt cotton flowers on the bollworm (*Helicoverpa zea*) with impacts to predation from a lady beetle (*Hippodamia convergens*)

**DOI:** 10.1371/journal.pone.0302941

**Published:** 2024-05-06

**Authors:** Blake H. Elkins, Maribel Portilla, Kerry Clint Allen, Nathan S. Little, Regina M. Mullen, Ryan T. Paulk, Quentin D. Read

**Affiliations:** 1 Southern Insect Management Research Unit, USDA-ARS, Stoneville, MS, United States of America; 2 Southeast Area, USDA-ARS, Raleigh, NC, United States of America; USDA Agricultural Research Service, UNITED STATES

## Abstract

Insecticidal *Bacillus thuringiensis* Berliner (Bt) toxins produced by transgenic cotton (*Gossypium hirsutum* L.) plants have become an essential component of cotton pest management. Bt toxins are the primary management tool in transgenic cotton for lepidopteran pests, the most important of which is the bollworm (*Helicoverpa zea* Boddie) (Lepidoptera: Noctuidae) in the United States (U.S.). However, bollworm larvae that survive after consuming Bt toxins may experience sublethal effects, which could alter interactions with other organisms, such as natural enemies. Experiments were conducted to evaluate how sublethal effects of a commercial Bt product (Dipel) incorporated into artificial diet and from Bt cotton flowers impact predation from the convergent lady beetle (*Hippodamia convergens* Guérin-Méneville) (Coleoptera: Coccinellidae), common in cotton fields of the mid-southern U.S. Sublethal effects were detected through reduced weight and slower development in bollworm larvae which fed on Dipel incorporated into artificial diet, Bollgard II, and Bollgard 3 cotton flowers. Sublethal effects from proteins incorporated into artificial diet were found to significantly alter predation from third instar lady beetle larvae. Predation of bollworm larvae also increased significantly after feeding for three days on a diet incorporated with Bt proteins. These results suggest that the changes in larval weight and development induced by Bt can be used to help predict consumption of bollworm larvae by the convergent lady beetle. These findings are essential to understanding the potential level of biological control in Bt cotton where lepidopteran larvae experience sublethal effects.

## Introduction

Cotton (*Gossypium hirsutum* L.) is an important fiber crop cultivated worldwide and is impacted by numerous insect pests [1,2]. Lepidopteran larvae are considered some of the most economically damaging. The bollworm (*Helicoverpa zea* Bodie) (Lepidoptera: Noctuidae) is the most prevalent lepidopteran pest of cotton in the United States (U.S.), which infested at least 60% of U.S. cotton acreage and required over $20 million in management costs in 2022 [3]. Insecticidal toxins produced by transgenic cotton expressing genes from the bacterium *Bacillus thuringiensis* Berliner (Bt) are the most prevalent form of pest management for Lepidoptera in cotton. Over 90% of cotton in the U.S. in 2022 expressed Bt toxins that target lepidopteran larvae [3]. Multiple Bt toxins target lepidopteran pests, with commercial transgenic cotton varieties in the United States expressing either two or three toxins. Dual toxin Bt cotton express some combination of Cry1Ac, Cry1Ab, Cry2Ab, Cry1F, or Cry2Ae. Bt cotton expressing three toxins includes a pair of the previous toxins in addition to Vip3A. Commercial dual toxin Bt cotton varieties have suffered from increasingly common failures in the field due to increased resistance of the bollworm to some Bt toxins [4,5]. However, resistance has not yet been widely reported from fields with cotton producing three toxins [6].

Bollworms and other lepidopteran pests that are not killed after feeding on Bt may still be negatively influenced by the toxins through sublethal effects. Previous research found bollworms may not be as susceptible to Bt toxins as other lepidopteran pests and can rapidly develop resistance in the field [5]. Sublethal effects of Bt toxins from plant tissue are well documented in the bollworm and have been found to alter growth, development, and fitness [7–9]. The occurrence of sublethal effects from Bt cotton could also be related to the variability of toxin concentration within fruiting structures of the cotton plant, over time, and as affected by environmental conditions [10–14]. Sublethal concentrations of Bt toxins in cotton flowers are especially concerning due to higher reported survival of bollworms feeding on these structures [15–18].

Insect natural enemies are crucial for pest management in row crops because they provide biological control services. Insect predators have been found to reduce bollworm densities and damage in maize [19,20], sorghum [21–24], and cotton [25–29]. Insect predators of the bollworm in cotton include about 60 species that primarily correspond to the orders Coleoptera, Hemiptera, and Neuroptera [30]. For example, Seagraves and Yeargan [19] found the spotted lady beetle (*Coleomegilla maculata* DeGeer) was responsible for a 26.1% reduction in sentinel bollworm eggs placed in sweet maize. In Bt cotton, natural enemies may be more numerous compared with cotton that exclusively uses synthetic insecticides to manage lepidopteran pests [31]. Studies evaluating the broad impacts of Bt proteins on natural enemy populations have shown mixed responses in the field [31–33]. However, sublethal effects of Bt toxins on bollworm larvae may alter natural enemy and pest interactions, which could impact predation and biological control in cotton fields. Multiple studies on sublethal effects have indicated there could be an impact on predation from natural enemies, but experimental evidence to directly link these is uncommon [9,34,35].

To evaluate the potential of Bt cotton to alter predation from natural enemies, a set of experiments was conducted to estimate sublethal effects of Bt on bollworm larvae and determine their impact on predation from the convergent lady beetle (*Hippodamia convergens* Guérin-Méneville) (Coleoptera: Coccinellidae). *Hippodamia convergens* is a voracious predator of bollworm eggs and early instar larvae and common in cotton fields across the mid-southern U.S. [28,30,36]. Bollworm larvae were exposed to Bt proteins by incorporation into an artificial diet and through feeding on Bt cotton flowers. Two developmental stages of lady beetle larvae were used across experiments to evaluate how lady beetle life stage influenced successful predation of bollworm larvae experiencing sublethal effects of Bt proteins and Bt cotton flowers.

## Material and methods

All insect colonies and experiments were conducted under laboratory conditions with 16 h: 8 h (light: dark), 25 ± 1°C, and 60 ± 10% relative humidity.

### Insect colonies

A bollworm colony has been continually reared at the United States Department of Agriculture (USDA) in Stoneville, MS for more than 50 years. This colony has been considered susceptible to Bt toxins, serving as a reference colony for studying Bt resistance in wild populations of bollworms [6,37]. Colony rearing procedures can be found in Gore et al. [38]. Bollworm larvae of the laboratory colony were reared individually on a nutrient-enriched soy wheat germ artificial diet [39]. This diet is considered optimal for heliothines (2.51% protein) and has been used in numerous studies to rear bollworm larvae in the laboratory [37,40–44]. Bollworm eggs and neonate larvae from the laboratory colony were available on an as-needed basis for all experiments.

A convergent lady beetle colony (*Hippodamia convergens*) was established by individuals collected from grain sorghum (*Sorghum bicolor* Moench) and cotton fields on a USDA research farm outside of Leland, MS. This colony was reared in the laboratory for approximately 4 months (~5 generations) prior to initiating experiments. Lady beetles were reared individually in small plastic cups (36.9 ml, T125-0090, SOLO CUP Co., Highland Park, IL). Adults were aggregated in a large container for 48 hours once per generation to mate. Lady beetles were fed a mixture of a general insect diet [45,46], frozen bollworm eggs from the laboratory colony, and bee pollen.

### Dipel assay

To study the sublethal effects of Bt proteins on the bollworm, larvae were reared with varying concentrations of a commercial Bt product (Dipel DF, Valent, BioSciences, Libertyville, IL) (Dipel). Dipel contains Cry-proteins, including those found in Bollgard II and Bollgard 3 cottons, in addition to bacterial spores and other synergists that specifically target the larvae of lepidopteran pests. Dipel has periodically been used by the USDA in Stoneville, MS to monitor the susceptibility of lepidopteran populations to Cry-proteins [37,47]. To establish the current susceptibility of the laboratory bollworm colony to Dipel, diet-incorporated bioassays were conducted with neonate larvae from the laboratory colony as described in Little et al. [37]. The concentrations of Dipel used in these preliminary bioassays were 0, 0.3, 1, 3, 10, 30, 100, and 300 μg/ml with 16 larvae per dose. Dipel was incorporated with the same nutrient-enriched soy wheat germ artificial diet used for rearing. Mortality of larvae were determined after seven days. The assay was replicated three times and from this initial test, two concentrations were determined and used in further assays to produce multiple levels of sublethal effects in bollworm larvae: LC_20_ (12.0 μg/ml) and LC_50_ (40.6 μg/ml).

Bollworm larvae were then reared on diet with three different concentrations of Dipel (diet treatments) to provide a range of sublethal effects. The base of all treatments was the artificial diet used in bollworm colony rearing. Diet treatments included untreated diet, diet incorporated with 12.0 μg/ml of Dipel (LC_20_), and diet incorporated with 40.6 μg/ml of Dipel (LC_50_). Approximately 4.5 ml of treated diet were added to clear plastic cups (36.9 ml, T125-0090, SOLO CUP Co., Highland Park, IL). After the diet cooled, a single neonate bollworm larva from the laboratory colony was placed in each cup. To produce enough surviving individuals from each diet treatment for a Dipel assay, approximately 150 bollworms were reared individually for each diet treatment.

At one, three, five, seven, and nine days after neonate bollworm larvae were placed on diet treatments, surviving bollworm larvae from each diet treatment were individually transferred to their own predation arenas. All bollworm larvae were weighed, and developmental instar was recorded prior to being placed in the predation arena. Bollworm instar was determined by measuring the number of molted head capsules, which are not consumed between instars [48]. Predation arenas consisted of a single clear polystyrene Petri dish (100mm x 15mm), which included a single moistened filter paper (55mm). After bollworm larvae were transferred to predation arenas, a single lady beetle larva (first or third instar) from the laboratory colony was placed within each arena. A single predation arena was considered an experimental unit. This entire experimental design was replicated three times. For the first replicate, there were 12 experimental units for each diet treatment (Untreated, LC_20_, and LC_50_) at each bollworm age timepoint (one, three, five, seven, and nine days) for each lady beetle instar (first and third instar). The second and third replicates included ten experimental units per treatment. However, five experimental petri dishes, that were damaged from the first instar lady beetle, day nine, LC_20_ diet treatment of replicate three, were excluded. Mortality of bollworm larvae within the predation arenas was evaluated after 24 hours. New bollworm larvae, lady beetle larvae, and predation arenas were used at each timepoint, which allowed each bollworm age to be considered independent.

### Cotton flower assay

The sublethal effects of Bt cotton flowers on bollworm predation were evaluated in a separate assay. Cotton plants were grown in a greenhouse located at the USDA in Stoneville, MS. Three commercial cotton varieties were used in this experiment, which included non-Bt (DP1822XF, Bayer CropScience, St. Louis, MO), Bollgard II (DP1646B2XF, Cry1Ac and Cry2Ab), and Bollgard 3 (DP2055B3XF, Cry1Ac, Cry2Ab, and Vip3A). Plants were fertilized (Miracle Grow Shake’n Feed All Purpose Plant Food, Miracle Grow Lawn Products, Marysville, OH) every two weeks and watered as needed. Greenhouse pests (whiteflies and spider mites) were managed using applications of Acetamiprid (Strafer Max, United Phosphorus, King of Prussia, PA) and Spiromesifen (Oberon 2SC, Bayer CropScience, St. Louis, MO) based on recommended rates from the Mississippi State Insect Control Guide [49]. Cotton plants were also treated with a growth regulator (Mepiquat chloride, Loveland Products, Inc., Morgantown, KY) on an ad hoc basis.

To generate enough surviving bollworm larvae from cotton flowers across all cotton varieties, 50 to 60 white cotton flowers from each cotton variety were collected from plants in the greenhouse and placed onto a 2% non-nutritive agar medium. This provided moisture and support for each cotton flower, within a clear plastic container (473 ml, MN16-0100, SOLO CUP Co., Highland Park, IL) fitted with a mesh lid. Within two hours of collecting cotton flowers from the greenhouse, a single neonate bollworm larva from the laboratory colony was placed into each cotton flower corolla. Because of the difficulty in obtaining survivors from Bt cotton flowers using a susceptible laboratory colony, the cotton flower assay was conducted for two bollworm age timepoints (one and three days). One and three days after neonates were placed in cotton flowers, five surviving larvae from five flowers of each cotton type were delicately removed. Each larva was weighed, instar was recorded, and individually placed into its own predation arena with a single lady beetle (first or third instar) larva from the laboratory colony, as in the Dipel assay. Bollworm instar was determined by counting the number of molted head capsules. Mortality of bollworm larvae within the predation arenas was recorded after 24 hours. Each cotton flower type (Non-Bt, Bollgard II, and Bollgard 3) by each bollworm age timepoint (one and three days) by each lady beetle instar (first and third instar) had five experimental units and the entire experimental design was replicated four times.

### Statistical analyses

To analyze the results from the Dipel assay for bollworm weights, a linear mixed model was developed to test the fixed effects of bollworm age (one, three, five, seven, and nine days), diet treatment (untreated, LC_20_, and LC_50_), and their interaction (R, v. 4.2.3, [50]; lme4 package, v. 1.1–31, [51]; emmeans package, v. 1.8.2, [52]; multcomp package, v. 1.4–20, [53]). Replicate, which functioned as a blocking factor, and predation arena identity within replicate were included as random intercepts in the model. Normal distribution of model residuals and homogeneity of variance were assessed graphically. Bollworm weight was log_10_(n+1) transformed to meet model assumptions. Kenward-Roger method was used to estimate the degrees of freedom. To analyze treatment effects on bollworm instar, a cumulative logistic mixed-effects model was fit with the same fixed and random effects as above. Because bollworm instar is an ordered categorical variable, it was modeled with an ordered multinomial response distribution and a cumulative logit link function. (ordinal package, v. 2022.11–16, [54]). To analyze bollworm mortality, a linear mixed model was developed to test the fixed effects of lady beetle instar (first and third), bollworm age (one, three, five, seven, and nine days), diet treatment (untreated, LC20, and LC50) and all two- and three-way interactions, with replicate and predation arena identity within replicate included as random intercepts. The bollworm mortality model used the binomial distribution with logit link function given that mortality was a binary response variable. For all models, significant differences between estimated means were evaluated using the Sidak correction for multiple comparisons. If a significant interaction between diet treatment and bollworm age or lady beetle instar was detected, mean separation was conducted between diet treatments at each level of the respective factor [55]. This was to evaluate how the effects of different sublethal doses of Dipel varied based on bollworm age or lady beetle instar.

To evaluate the relationships between response variables from the Dipel assay (bollworm mortality, weight, and instar), univariate logarithmic regression was used. Bollworm weight and instar each acted as an independent variable. Predicted mortality served as the dependent variable in both cases. Model statistics, including calculated F, P, and R^2^ values, were provided to estimate how bollworm weight and instar performed as predicators for bollworm mortality from predation for first and third instar convergent lady beetles.

For the cotton flower assay, a linear model was used to analyze bollworm weights with fixed effects of bollworm age (one and three days), cotton flower type (Non-Bt, Bollgard II, and Bollgard 3 flowers), and the two-way interaction (same statistical packages as the Dipel assay models). Because of the difficulty of obtaining survivors from Bt cotton flowers (not all replicates had a single surviving larvae on day three from either Bt cotton type), all replicates were combined as in a completely randomized design. Weight was log_10_(n+1) transformed as in the analysis of the Dipel assay. A similar model was used for bollworm instar (same fixed effects and interaction), which used the binomial distribution with logit link function given that this was binary variable. To analyze bollworm mortality in the cotton flower assay, a linear model was developed to test the fixed effects of lady beetle instar (first and third), bollworm age (one and three days), cotton flower type (Non-Bt, Bollgard II, and Bollgard 3 flowers), and all two- and three-way interactions. This model used the binomial distribution with logit link function given that mortality was a binary variable. Significant differences between estimated means were evaluated using the same approach as the Dipel assay. If a significant interaction between cotton type and bollworm age or lady beetle instar was detected, mean separation was conducted between cotton types at each level of the respective factor [55]. This was to evaluate how the effects of Bt and non-Bt cotton flowers could vary based on bollworm age or lady beetle instar.

## Results

### Dipel assay

Significant sublethal effects of Dipel were observed through the reduced weights and instars of bollworm larvae from both the LC_20_ and LC_50_ diet treatments (Table 1). These sublethal effects were significantly different across diet types for weights (F = 1967.03; d.f. = 2, 909.18; P < 0.001) and instar (G = 1572.70; d.f. = 13; P < 0.001). They also significantly differed across bollworm age treatments for weight (F = 1257.58; d.f. = 4; 909.18; P < 0.001) and instar (G = 832.33; d.f. = 11; P < 0.001). There was a significant interaction of these effects for bollworm weight (F = 209.85; d.f. = 8, 909.18; P < 0.001) and instar (G = 23.27; d.f. = 1; P < 0.001), with diet types not significantly different at day one and both Dipel treatments significantly less than untreated diet at day three. Also, by day five, the LC_50_ diet had significantly reduced weight and development compared to the LC_20_ diet (Table 1).

**Table 1:** Mean (± S.E.) bollworm weight (mg), instar, and number of larvae tested (n) across all replicates by bollworm age (days) and Dipel treatments incorporated into artificial diet. Data were combined across lady beetle instars. Different letters designate significant (P ≤ 0.05) differences between diet treatments within a bollworm age timepoint.

Bollworm Age (Day)	Bollworm Diet Treatment	n	Bollworm Weight (mg)	Bollworm Instar
1	Untreated	64	0.33 ± 0.03a	1.00 ± 0.00a
	LC_20_	64	0.18 ± 0.02a	1.00 ± 0.00a
	LC_50_	64	0.13 ± 0.01a	1.00 ± 0.00a
3	Untreated	64	2.26 ± 0.15a	1.84 ± 0.05a
	LC_20_	64	0.45 ± 0.04b	1.08 ± 0.03b
	LC_50_	64	0.30 ± 0.03b	1.05 ± 0.03b
5	Untreated	64	13.49 ± 0.79a	2.96 ± 0.04a
	LC_20_	64	1.29 ± 0.11b	1.87 ± 0.04b
	LC_50_	64	0.81 ± 0.12c	1.53 ± 0.06c
7	Untreated	64	48.01 ± 2.26a	3.95 ± 0.06a
	LC_20_	64	3.41 ± 0.39b	2.39 ± 0.06b
	LC_50_	64	2.32 ± 0.40c	2.02 ± 0.05c
9	Untreated	64	129.50 ± 6.36a	4.08 ± 0.06a
	LC_20_	59*	11.10 ± 1.59b	3.00 ± 0.05b
	LC_50_	64	4.00 ± 0.89c	2.56 ± 0.06c

* five experiment units from this treatment were damaged and excluded.

**Fig 1:**
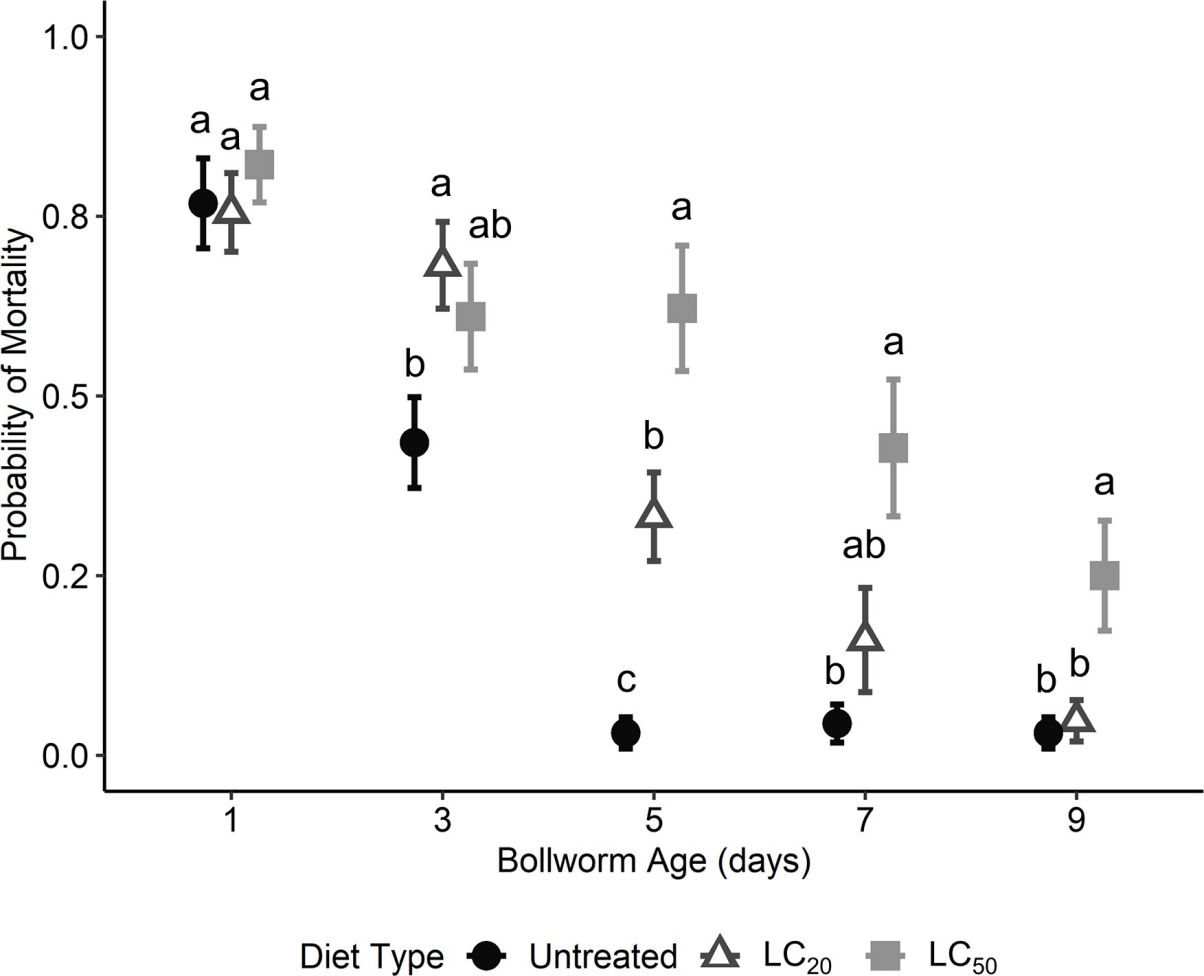
Predicted probability of mortality of bollworm larvae from lady beetle larvae when reared individually on untreated artificial diet, diet with 12.0 μg/ml of Dipel (LC_20_), or 40.6 μg/ml of Dipel (LC_50_) over time. Different letters designate significant (P ≤ 0.05) differences between diet treatments within a bollworm age timepoint.

**Fig 2:**
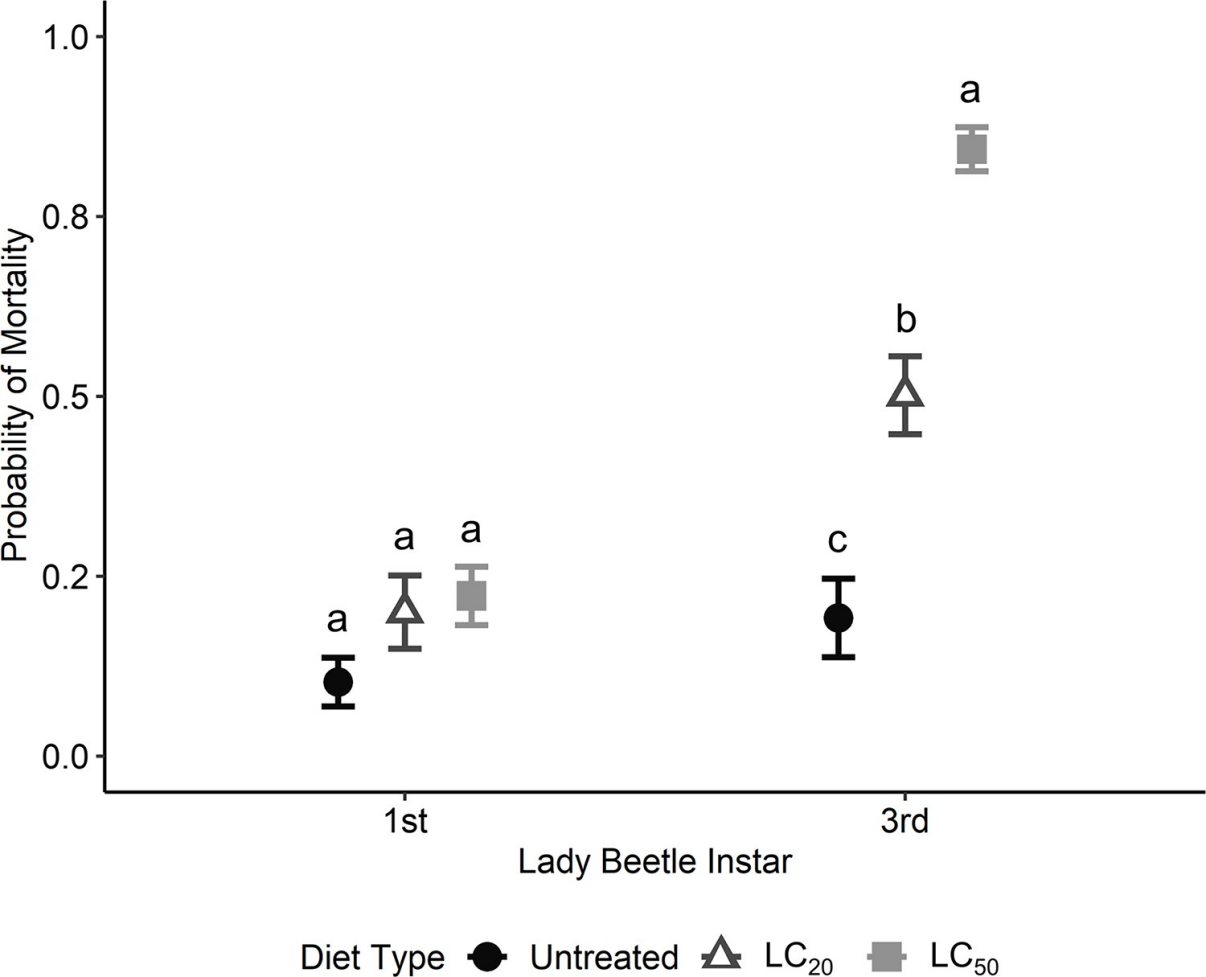
Predicted probability of mortality of bollworm larvae from first and third instar lady beetle larvae when reared individually on untreated artificial diet, diet with 12.0 μg/ml of Dipel (LC_20_), or 40.6 μg/ml of Dipel (LC_50_). Different letters designate significant (P ≤ 0.05) differences between diet treatments within a lady beetle instar.

Diet and bollworm age treatments, which displayed significant sublethal effects on bollworm weights and instars, also had significant effects on bollworm predation. There was a significant effect of bollworm age (χ^2^ = 138.94; d.f. = 4; P < 0.001), diet treatment (χ^2^ = 36.66; d.f. = 2; P < 0.001), and lady beetle instar (χ^2^ = 80.42; d.f. = 1; P < 0.001) on bollworm mortality. The interaction between bollworm diet and bollworm age was significant (χ^2^ = 26.58; d.f. = 8; P = 0.001). The LC_20_ diet treatment had significantly greater bollworm mortality from first and third instar lady beetle larvae combined compared to the untreated diet on days three and five (Fig 1). Bollworm morality on the LC_50_ diet was significantly greater than the untreated diet at days five, seven, and nine (Fig 1). Additionally, bollworm mortality in the LC_50_ diet was significantly greater than the LC_20_ treatment on days five and nine (Fig 1). There was also a significant interaction between bollworm diet and lady beetle instar (χ^2^ = 15.25; d.f. = 2; P < 0.001) (Fig 1). Differences in mortality between diet treatments for first instar lady beetles were not significant (Fig 2). For third instar lady beetles, bollworm mortality on the LC_50_ diet was significantly greater than the LC_20_ diet, which was significantly greater than the untreated diet (Fig 2). There was no significant interaction between bollworm age and lady beetle instar (χ^2^ = 5.72; d.f. = 4; P = 0.221) or the three-way interaction (χ^2^ = 11.17; d.f. = 8; P = 0.192).

**Fig 3:**
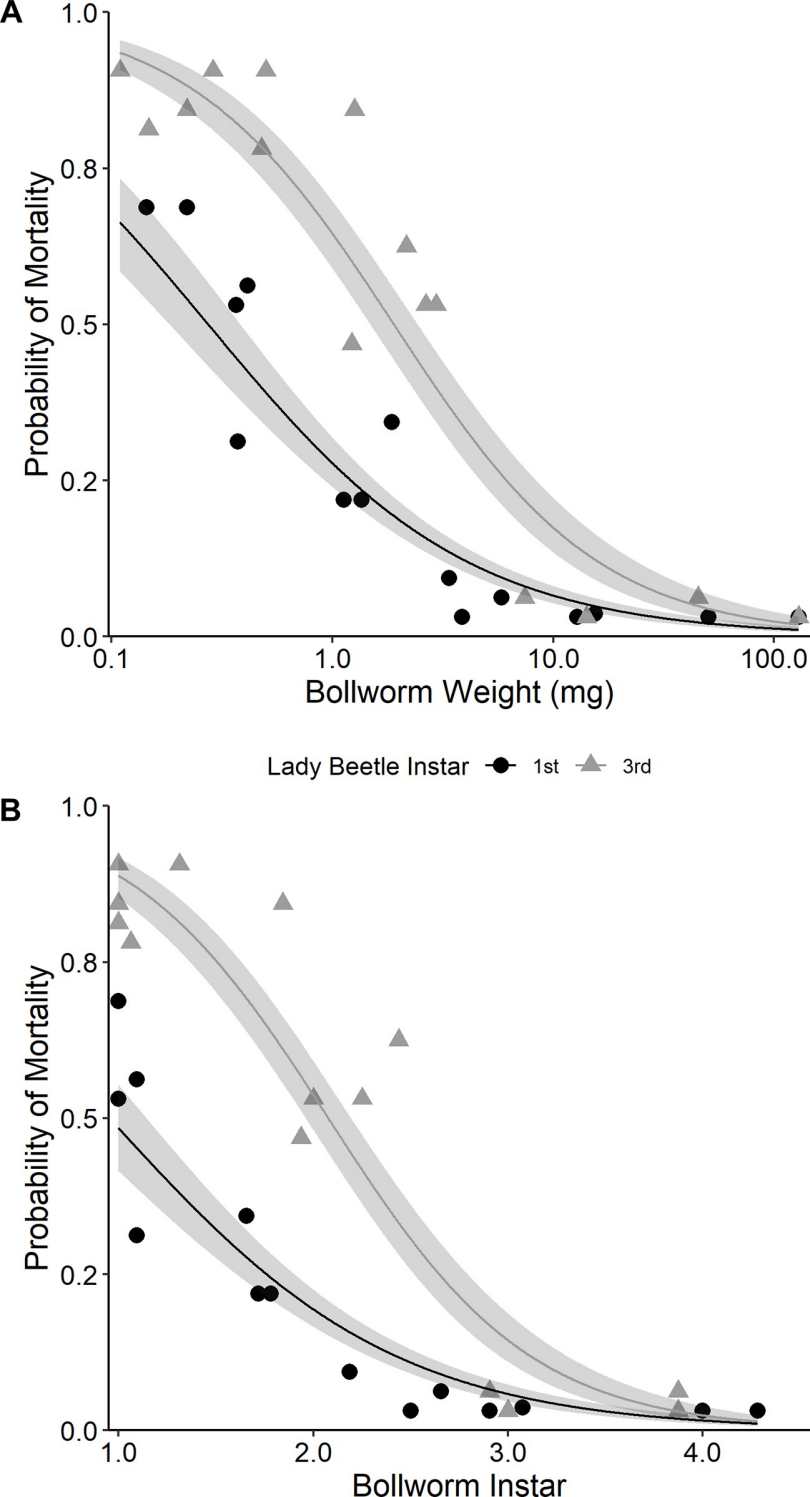
Relationship (logarithmic) between predicted probability of mortality of bollworm larvae and (A) bollworm weight (mg) or (B) bollworm instar from the Dipel experiment. Shaded areas indicate the 95% confidence intervals.

Bollworm weight and instar served as predictors of the probability of mortality from predation. Bollworm weight (Fig 3A) predicted 82% of the variation in the probability of mortality for first instar lady beetle larvae (F = 64.54; d.f. = 1, 13; P < 0.001; R^2^ = 0.820) and 83% of the variability for the third instar lady beetle larvae (F = 68.69; d.f. = 1, 13; P < 0.001; R^2^ = 0.829). Bollworm instar (Fig 3B) was similar, which predicted 80% of the variation in the probability of mortality for first instar lady beetle larvae (F = 57.37; d.f. = 1, 13; P < 0.001; R^2^ = 0.801) and 84% of the variability for third instar lady beetle larvae (F = 72.84; d.f. = 1, 13; P < 0.001; R^2^ = 0.837).

### Cotton flower assay

**Fig 4:**
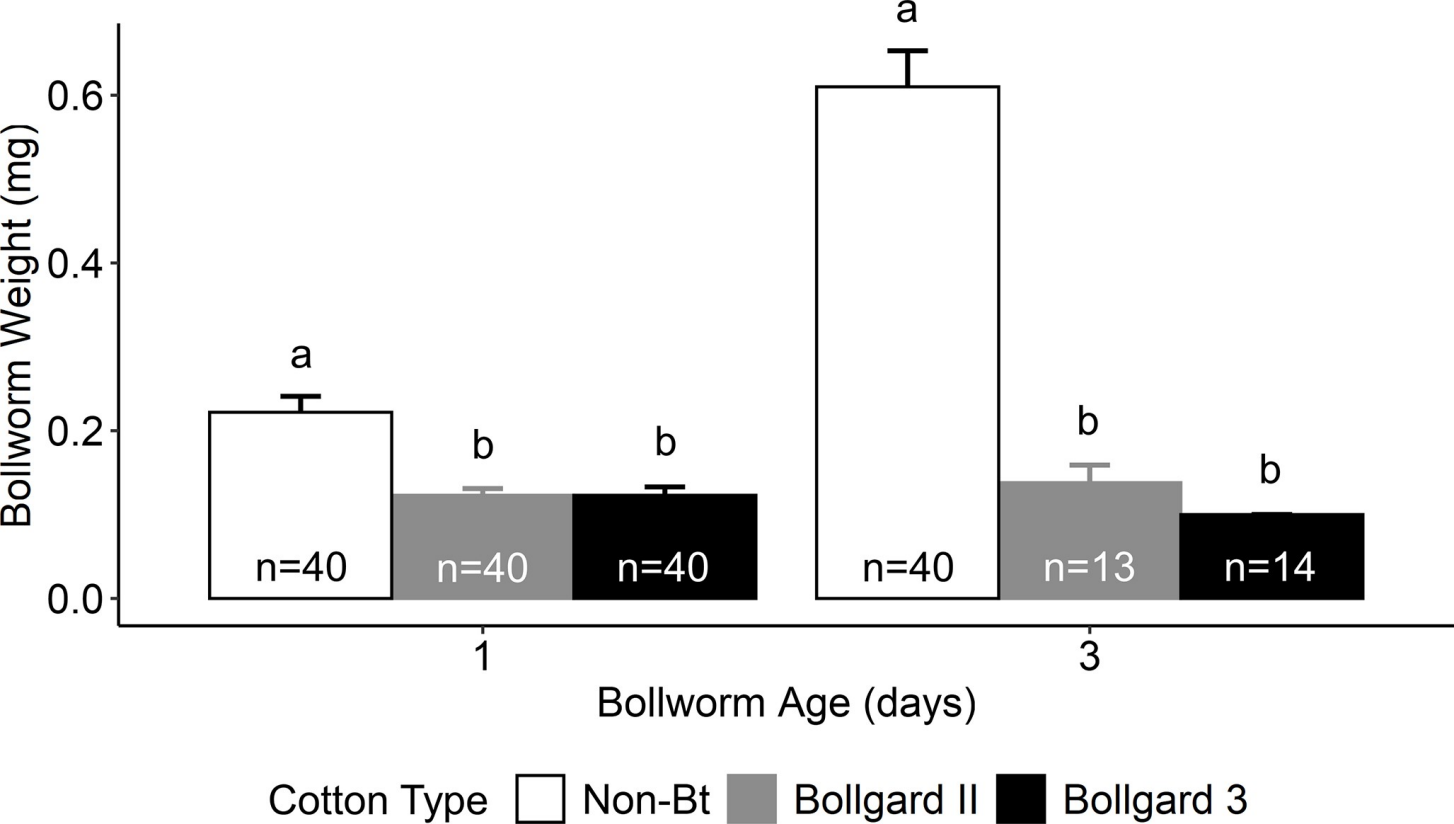
Mean (± S.E.) bollworm weight (mg) and number of larvae tested (n) across all replicates by bollworm age (days) and cotton flower treatment. Data were combined across lady beetle instars. Different letters designate significant (P ≤ 0.05) differences between means within a bollworm age timepoint.

Survival of neonate bollworm larvae in non-Bt cotton flowers was generally high (> 90%) for non-Bt cotton flowers and low (< 10%) for Bt cotton flowers. This contributed to a reduced number of individuals tested from Bollgard II and Bollgard 3 cotton flowers for day three of the cotton flower assay (Fig 4). Similar to the results from the Dipel assay, analyses indicated that bollworm age significantly affected bollworm weight (F = 157.42; d.f. = 1, 181; P < 0.001) and instar (χ^2^ = 79.51; d.f. = 1; P < 0.001). Cotton flower type also significantly affected bollworm weight (F = 82.37; d.f. = 2, 181; P < 0.001) and instar (χ^2^ = 62.61; d.f. = 2; P < 0.001). There was a significant interaction between cotton flower type and bollworm age for bollworm weight (F = 41.97; d.f. = 2, 181; P < 0.001). Bollworm weight more than doubled from day one to three for non-Bt, but not for Bt cotton flowers (Fig 4). Larval weight was significantly lower in Bollgard II and Bollgard 3 compared to non-Bt for days one and three, with no significant differences in sublethal effects observed between Bollgard II and Bollgard 3 during this three-day assay (Fig 4). The interaction of cotton flower type and bollworm age was not significant for instar (χ^2^ < 0.001; d.f. = 2; P = 1.000). Bollworm instar was greater in non-Bt cotton flowers (1.44 ± 0.06) compared to bollworms from Bollgard II and Bollgard 3, which did not develop beyond first instar. Similarly, all bollworm larvae were first instar on day one, but by day three, the average instar had significantly increased (1.52 ± 0.06).

**Fig 5:**
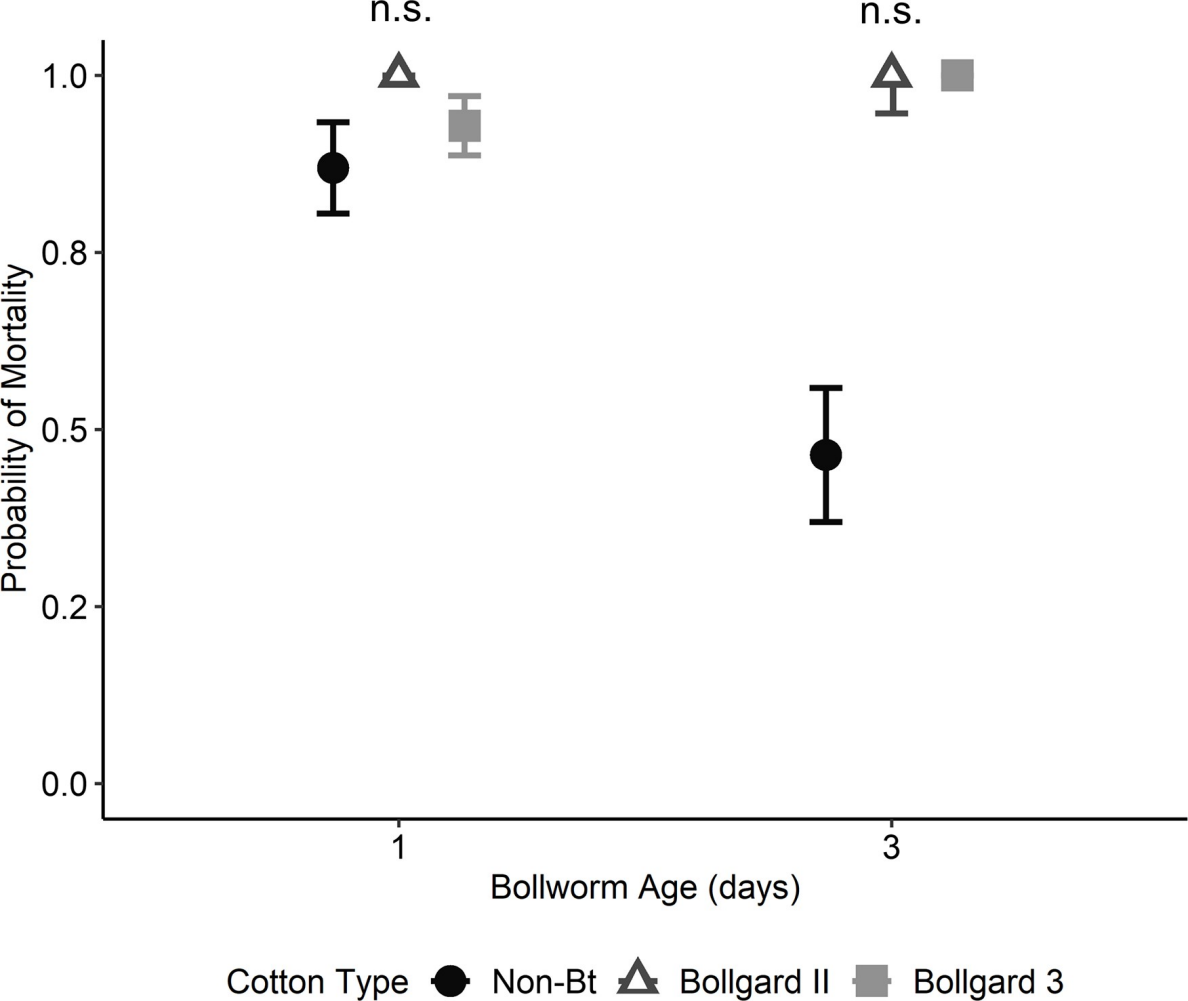
Predicted probability of mortality of bollworm larvae from first and third instar lady beetle larvae when reared individually on non-Bt, Bollgard II, or Bollgard 3 cotton flowers. N.S. designates no significant differences between cotton flower types within a bollworm age.

The probability of mortality from predation in the cotton flower assay was significantly impacted by bollworm age (χ^2^ = 10.60; d.f. = 1; P = 0.001), cotton flower type (χ^2^ = 26.89; d.f. = 2; P < 0.001), and lady beetle instar (χ^2^ = 14.61; d.f. = 1; P < 0.001), with a significant interaction between bollworm age and cotton flower type (χ^2^ = 7.75; d.f. = 2; P = 0.021). All other two-way and the three-way interactions were not significant (P ≥ 0.142). Although estimated bollworm mortality from predation was greater in Bt (100%) than non-Bt cotton flowers (71%), there were no significant differences between cotton flower types at any bollworm age after adjusting for multiple comparisons (Fig 5).

## Discussion

This study found that bollworm larvae feeding on sublethal concentrations of Bt proteins alters predation from the convergent lady beetle. The Dipel assay demonstrated that the level of sublethal effects (diet type and bollworm age) and the growth stage of the natural enemy (lady beetle instar) impact predation of bollworm larvae. These impacts included greater predation associated with higher concentrations of Dipel, less developed bollworm larvae, and more developed lady beetle larvae. Furthermore, the LC_50_ diet treatment in the Dipel assay showed significantly higher mortality at days five through nine compared to the untreated control. This demonstrates how the limited temporal window in which a convergent lady beetle is able to consume a bollworm larva can be extended by the sublethal effects of Bt. Prey size has previously been acknowledged as a predictor of the outcome of predator-prey interactions [56]. For lady beetles specifically, the difference in size between predator and prey is crucial for determining a lady beetle’s ability to consume a specific prey item [57,58]. Other studies have demonstrated how predation of lepidopteran larvae decreased as larval size and development increased. For example, predation of tobacco budworm (*Chloridea virescens* F.) by the big-eyed bug (*Geocoris punctipes* Say) decreased as larval development and size increased until there was no predation once larvae had developed to third instar [59,60]. As lady beetle larvae were able to consume 7.1% of third instar bollworm larvae from Bt treatments and 2.6% from the non-Bt treatment in this study, sublethal effects from Bt may improve consumption of bollworm larvae for other natural enemies that are limited to a certain developmental instar or size on non-Bt cotton. This could expand the potential complex of predatory species capable of suppressing bollworm larvae in Bt cotton by allowing them to feed on bollworm larvae that would have otherwise developed beyond the predatory capabilities of the natural enemies.

Sublethal effects of Bt on lepidopteran larvae and their ability to impact predation by natural enemies has been a topic of interest in the literature since before the commercial introduction of Bt crops [61,62]. The research presented demonstrates how bollworm larvae can experience sublethal effects from a commercial formulation of Bt incorporated into artificial diet and from flowers of Bollgard II and Bollgard 3 cottons. Decreased larval weights were observed in as little as one day in the cotton flower assay and by day three in the Dipel assay. Delayed larval development was observed by day three for both assay types. Additionally, bollworm larvae in the LC_50_ treatment had a reduced weight and development compared to the LC_20_ treatment by day five. The findings of reduced weight and development of larvae after consuming Bt are consistent with other studies that have evaluated the sublethal effects of different Bt toxins on lepidopteran larvae [7–9]. While the Cry-proteins in Bollgard II and Bollgard 3 cotton were also present in Dipel, the effect of these different forms of Bt on bollworm larvae and predation were not always consistent. This indicates that something other than the presence of a Bt protein determine the level of sublethal effects and the impact to predation. Other differences such as the relative concentration or source of Bt may have mattered more.

Sublethal effects of Bt on bollworm larvae could have other impacts besides delaying growth and development that might influence predation. While lepidopteran larvae can exhibit defensive behaviors following hatching, bollworms are considered more aggressive while interacting with conspecifics compared to other lepidopteran species, such as the fall armyworm (*Spodoptera frugiperda* J.E. Smith) and tobacco budworm [63–65]. Previous studies have shown that aggressive behaviors of the bollworm tend to increase over larval development and sublethal effects that delay development could also delay aggressive behavior making them more susceptible to predation [63,65]. Studies have also demonstrated modified behavior of bollworm larvae consistent with enhanced survival in Bt cotton, such as egg and larval distribution within the canopy, which could alter predation from natural enemies [66–68]. Linking sublethal effects to the potential of biological control has relevant implications for pest management because understanding the ability of an individual natural enemy to consume bollworm larvae will influence the level of suppression able to be achieved in the field [69].

This experiment found that significant sublethal effects of Bt cotton flowers were not translated into significant differences in bollworm mortality within the predation arenas. This may have been due to the high mortality of bollworm larvae in Bollgard II and Bollgard 3 cotton flowers, which resulted in a low sample size. The high mortality of bollworm larvae was the reason the cotton flower assay was not taken beyond the three-day bollworm age timepoint, which was when significant differences appeared in the Dipel assay. The mortality of bollworm larvae in Bt cotton flowers, especially Bollgard II, exceeded original expectations of mortality in cotton flowers, as flowers were expected to have reduced toxin concentrations and lower mortality compared to other fruiting structures [15–17]. For example, Godbold et al. [18] found less than 50% mortality of neonate bollworm larvae using a field colony after three days feeding on excised flowers from Bollgard II and Bollgard 3 cottons. Additionally, nutritional differences between cotton structures and varieties may alter bollworm survival and fitness leading to variable responses to Bt across different cotton plants [70]. The unexpectedly high mortality was in part due to the use of a laboratory colony known to have high susceptibility to Bt toxins [37]. However, susceptible colonies may be considered more appropriate for representing the sublethal effects of Bt toxins [71].

This research demonstrated how bollworm larvae feeding on sublethal concentrations of Bt proteins can influence predation from a common natural enemy in cotton. The results suggest that sublethal effects of Bt could synergize with the direct mortality caused by Bt proteins in the presence of certain natural enemies, enhancing biological control. Similar positive or neutral indirect interactions between Bt and natural enemies have been found in other studies [56,57,72,73]. This would also help explain other reports of synergistic interactions between Bt and natural enemies such as the delayed development of resistance to Bt toxins [74]. Lopez et al. [75] found that while the spotted lady beetle could consume around 104 first instar tobacco budworm larvae per day, it could only consume around 0.5 third instar larvae per day. This led the researchers to conclude that the spotted lady beetle was a poor predator of third instar larvae. Results from the present study demonstrate how the ability of a lady beetle to consume more developed bollworm larvae could be increased given sublethal Bt exposure.

An important consideration to the application of these findings to biological control in Bt cotton fields is the complex relationship between Bt cotton, sublethal effects, and natural enemies. For example, natural enemies that consumed prey experiencing sublethal effects from Bt toxins had reduced fitness and may have contributed to reduced natural enemy populations in Bt crop fields [31–33]. We did not evaluate predator choice in this experiment, which likely has important implications for biological control and should be a focus of future studies. These findings still demonstrate that sublethal effects of bollworm larvae feeding on Bt toxins may have an impact on pest suppression from natural enemies observed in transgenic cotton and should be considered for future investigations in the field.

## Supporting information

S1 Data (XLSX)
